# Strain-related variation in the persistence of influenza A virus in three types of water: distilled water, filtered surface water, and intact surface water

**DOI:** 10.1186/1743-422X-10-13

**Published:** 2013-01-07

**Authors:** Shamus P Keeler, Camille Lebarbenchon, David E Stallknecht

**Affiliations:** 1 Southeastern Cooperative Wildlife Disease Study, Department of Population Health, College of Veterinary Medicine, The University of Georgia, Athens, GA 30602, USA; 2 Department of Infectious Diseases, College of Veterinary Medicine, University of Georgia, Athens, GA 30602, USA; 3 Current address: Université de la Réunion, Avenue René Cassin, BP 7151, 97715, Saint-Denis cedex, La Réunion, France

## Abstract

**Background:**

The persistence of influenza A (IA) virus in aquatic habitats has been demonstrated to be a determinant for virus transmission dynamics in wild duck populations. In this study, we investigated virus strain-related variation in persistence in water for nine wild duck isolated IA viruses of three subtypes (H3N8, H4N6, and H8N4).

**Results:**

We experimentally estimated the loss of infectivity over time in three different types of water: distilled, filtered surface water, and intact surface water. All viruses persisted longest in distilled water followed by filtered surface water with markedly reduced durations of persistence observed in the intact surface water. Strain-related variations were observed in distilled and filtered surface water but limited variation was observed in the intact surface water.

**Conclusions:**

Our findings suggest that the role of surface water for long-term (between years) maintenance of AI viruses in the environment may be limited, and suggest that the physico-chemical characteristics of water, as well as microorganisms, may be of strong importance. Results also indicate that the extent of strain-related variation observed in distilled water may overestimate persistence abilities for IA viruses in the wild and supports the need to develop experiments that account for these effects to assess subtype, genotype, as well as spatial and temporal variation in the persistence of IA viruses in aquatic habitats.

## Background

Influenza A (IA) virus persistence in aquatic habitats has been demonstrated to be a determinant for virus transmission dynamics in wild duck populations [1-3]. In these hosts, viral replication mainly occurs in the epithelial cells of the intestinal tract, resulting in high virus concentration in feces [4,5]. Infected birds contaminate aquatic environments in which IA viruses can persist for extended periods of time, depending on water temperature and physico-chemical characteristics [6-11]. Biotic components including aquatic invertebrates and microorganisms also have recently been proposed as potential factors affecting virus removal or accumulation in the environment [10,12-14].

Strain-related variations in the persistence of IA viruses have been investigated under experimental conditions using distilled water maintained at different temperatures, pH, and salinity [8,15]. In a recent study, Lebarbenchon et al. [16] suggested that differences in the persistence of IA viruses in water may be limited when considering co-circulating viruses in a single duck population. However, variation in the duration of persistence has been documented for viruses circulating in different locations or seasons [8], suggesting potential adaptive responses of IA viruses to local water characteristics and the ability to rapidly evolve toward an optimal level of persistence in changing environments [16].

While studies have compared the aquatic stability of multiple IA virus strains, to date, these studies have been limited to comparisons of persistence in distilled water [8,15,16]. Direct comparison of the duration of persistence of viruses under more realistic conditions are limited, in particular the effects of the physico-chemical characteristics and microorganisms in surface water have remained poorly understood. In this study, we investigated virus strain-related variation for three common IA virus subtypes in wild duck populations: H3N8, H4N6, and H8N4 [17-19]. We experimentally estimated the loss of infectivity over time of nine virus strains, in three different types of water: distilled water, filtered surface water, and intact surface water. We discuss results in the light of current knowledge on IA virus ecology in wild duck populations and the role for water-borne transmission in avian influenza epidemiology.

## Results

**Figure 1:**
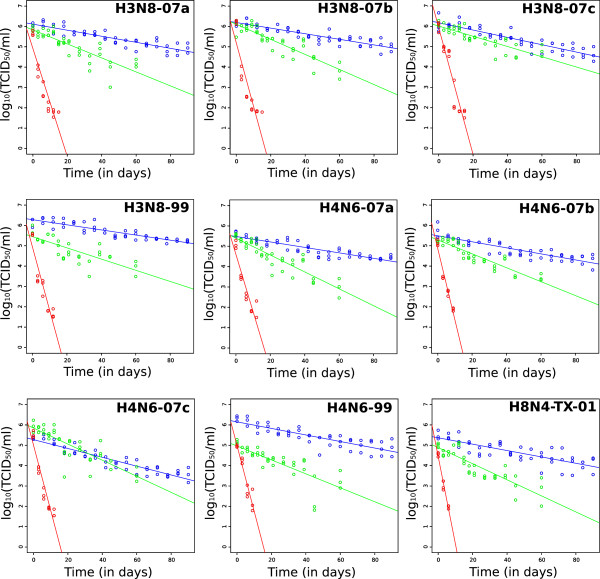
**Virus infectivity in water over time as a function of water type.** Color lines represent the least-squares regression lines for each water type (blue: distilled water; green: filtered surface water; red: intact surface water).

Viral persistence in water significantly decreased over time (F_1,856_ = 75, P < 0.001), with evidence that this decrease strongly varied with the type of water (time by water type interaction: F_2,856_ = 937, P < 0.001). The main effect of water type was also significant (F_2,856_ = 2392, P < 0.001): all virus strains persisted longest in distilled water followed by filtered surface water with markedly reduced duration of persistence observed in intact surface water (Table 1, Figure 1). The effect of virus on persistence was significant (F_8,856_ = 88, P < 0.001); however, this effect may be induced by slight differences in the initial doses used for each virus. A strong interaction was found between the time and the viral strain (F_8,856_ = 21, P < 0.001), indicating that the decrease in virus titer over time varied among viruses. Also, a significant interaction between the water type and the viral strain was found (F_16,856_ = 21, P < 0.001) suggesting that the pattern of infectivity loss induced by the water type differed between viruses. Finally, there was a significant three way interaction between time, virus, and water type (F_16,856_ = 2.3, P < 0.01), indicating that the effect of the water type on viral persistence over time was different among virus strains.

**Table 1:** Summary of the average virus log reduction times

	**Water Type**		
**Virus Strain**	**Distilled**	**Filtered Surface**	**Intact Surface**
H3N8-07a	65.9 (3.7)	29.2 (3.1)	3.7 (0.4)
H3N8-07b	73.7 (10.8)	27.1 (1.5)	3.0 (0.8)
H3N8-99	78.8 (2.9)	36.9 (5.9)	3.2 (0.1)
H3N8-07c	59.1 (14.2)	41.4 (10.7)	3.2 (0.1)
H8N4-TX-01	68.6 (18.8)	26.2 (6.2)	2.3 (0.1)
H4N6-07a	75.6 (2.3)	24.4 (3.1)	3.5 (0.8)
H4N6-07b	69.2 (14.4)	29.3 (2.3)	2.8 (0.1)
H4N6-99	66.6 (17.2)	29.9 (5.3)	3.2 (0.7)
H4N6-07c	46.2 (2.1)	25.6 (3.2)	3.1 (0.2)
Average	67.1 (9.8)	30.0 (5.6)	3.1 (0.4)

Average virus log reduction times (Rt) with standard deviation in parentheses for each virus strain and water type combination. The Rt values are the time (days) required for a decrease of viral titer by 1 log_10_ TCID_50_/ml.

To further investigate differences in the pattern of loss of infectivity over time we performed an independent ANCOVA for each water type. The main effects of time and virus were significant for all water types. A significant interaction between time and virus strain was found for the distilled (F_8,414_ = 4.96, P < 0.001) and filtered (F_8,333_ = 2.47, P < 0.05) water. For the intact surface water however, this interaction was no longer significant (F_8,109_ = 0.88, P = 0.53). H4N6-07c exhibited a significantly higher loss of infectivity as compared to other virus strains, in distilled water, however a note of caution is warranted since a lower dose of virus was used for this virus.

## Discussion

All viral strains showed a markedly reduced duration of persistence in intact surface water compared to both filtered surface water and distilled water. The surface water sample we used had pH and salinity levels considered to be ideal for long-term persistence (i.e. neutral to basic pH and low salinity [8]) and the trials were conducted at a constant incubation temperature (17°C), limiting previously documented temperature-related effects [11,16]. These results are consistent with previous studies involving intact surface water [10,12]; the duration of IA virus persistence in the surface water of aquatic habitats can significantly be reduced by adverse physico-chemical conditions [20] or due to the presence of a wide variety of biological organisms including bacteria, fungi, algae, and protozoa [12]. The duration of persistence in the filtered surface water used in our experiments was significantly increased (ten-fold) as compared to intact surface water, suggesting that microorganisms or other nonorganic particulate matter present in the water sample could limit the ability of IA virus to remain infectious in aquatic habitats. The surface water sample used in this study was not biologically characterized. The pond that was sampled is located in an urban area inside a public park and the site could have higher microorganism counts compared to more pristine (less human impacted) ecosystems [21,22]. If microorganisms are reducing the environmental stability of the virus, surface water with higher microbial counts maybe less hospitable to viral persistence so the degree of reduction of viral persistence observed in this study may not be universally applicable to all surface water. Overall, the reduction in the duration of persistence observed in intact surface water suggests that it may not represent a suitable environment for long-term maintenance (between years) of IA viruses as was suggested based on persistence trials performed entirely in modified distilled water. The importance of other components of aquatic ecosystems (e.g. soil [23,24], aquatic invertebrates [14]) to virus persistence and to the transmission dynamics of IA viruses in wild duck population, remain poorly understood. These factors may however favor long-term (between-years) maintenance of viruses in the environment, in particular between epidemics, and require further investigation.

Consistent with previous studies involving multiple IA viruses, variation in the duration of viral persistence was observed between strains in distilled water and even in filtered surface water [8,15]. The strain-related differences were considerably reduced in the intact surface water indicating that viral strain or subtype may only have a limited effect on the persistence of viruses in surface water. These findings suggest that usually reported strain-related variations in the duration of infectivity in distilled water may not reflect realistic persistence abilities for IA viruses at least at the temperature evaluated in this study. Future experimental designs need to consider this aspect to evaluate potential subtype, genotype as well as spatial and temporal variation in the persistence of these viruses in aquatic habitats.

The viruses used in this study were isolated from waterfowl congregating in large numbers as part of a seasonal migratory pattern [19,25]. During these times of mass gatherings of birds, viral adaptation to local water conditions would not be necessary as the majority of the transmission would be driven by the density of birds occupying the site [3]. The viruses we selected for this project would have only required a minimal amount of environmental persistence to ensure indirect transmission during these periods of high bird density and this could explain why limited strain-related differences were observed in intact surface water but further studies are required to evaluate the validity of this hypothesis.

The results of this study provide further insight into the role of surface water as a medium for virus transmission, but limited evidence that surface water could act as long-term environmental reservoir for IA viruses. Strain related differences in virus persistence were not observed in intact-surface water indicating that water physico-chemical characteristics as well as microorganisms may significantly reduce the duration of persistence of IA viruses in water. The intact surface water used in this study was collected from a single source, which limits the universality of the conclusions of the study but the findings support the need to develop experimental designs reflecting natural conditions in order to better assess strain-related variation in the maintenance of IA virus in aquatic environments.

## Methods

### Virus selection

All viruses were isolated from dabbling duck species. Six viruses were obtained from a population surveillance study in Minnesota conducted in 2007 [19]: A/Mallard/Minnesota/Sg-00051/2007 (H3N8), A/Mallard/Minnesota/Sg-00048/2007 (H3N8), A/Mallard/Minnesota/Sg-00169/2007 (H3N8) (referred to hereafter as H3N8-07a, H3N8-07b, and H3N8-07c); and A/Mallard/Minnesota/Sg-00050/2007 (H4N6), A/Mallard/Minnesota/Sg-00053/2007 (H4N6), A/Mallard/Minnesota/Sg-00063/2007 (H4N6) (referred to hereafter as H4N6-07a, H4N6-07b and H4N6-07c). Two additional viruses isolated in Mallards in Minnesota in 1999 were included: A/Mallard/Minnesota/199106/1999 (H3N8) and A/Mallard/Minnesota/199044/1999 (H4N6) (referred to hereafter as H3N8-99 and H4N6-99). Finally, a H8N4 virus isolated in Texas in 2001 was also included: A/Northern Pintail/TX/421716/01 (referred to hereafter as H8N4-TX-01).

Stock viruses were propagated in 9 to 11 day old specific pathogen free (SPF) embryonating chicken eggs with all viruses being second passage [26]. Serial titrations were performed in SPF embryonating chicken eggs and Madin Darby canine kidney (MDCK) cells to determine the median embryo infectious dose (EID_50_) and the median tissue culture infectious dose (TCID_50_) [26,27].

### Water-persistence trials

For each of the nine viruses, we tested the effect of the water type (distilled, filtered surface, and intact surface), on the decrease in infectivity over time. A surface water sample was collected from Memorial Pond (33°55'37.31"N, 83°23'2.71"W), a 12,141 m^3^ man-made lake inside a recreational park, on 06-May 2011. The site was selected for convenience as the site is close to the lab, and the pond was known to have a neutral pH and low salinity. The pond also has resident waterfowl species including peridomestic muscovy ducks (*Cairina moschata*) and mallards (*Anas platyrhynchos*). Three 1 L water samples were collected in LDPE wide-mouth bottles (Thermo Fisher Scientific, Inc, Waltham, Massachusetts, USA) within 1 m of the shoreline and about 3 cm below the surface. Samples were placed on ice for transport back to the lab. Specific conductance and pH readings were taken at the site of collection using a YSI 556 MPS handheld instrument (YSI, Inc, Yellow Springs, Ohio, USA). In the lab, half of each 1 L water sample was filtered using a bottle-top vacuum filter system with a 0.22 μm polyethersulfone membrane (Corning Inc, Corning, New York, USA) to remove most biological material and the other half was maintained intact. The pH was confirmed in the laboratory for both the filtered and intact surface water using a VWR sympHony SB80PC bench top meter (VWR International, Radmor, Pennsylvanian, USA). The average pH for all surface water samples was determined to be 7.2 and the salinity was 12 parts per million (ppm).

Distilled water buffered with 10 mM HEPES was adjusted with 1 N solutions of NaOH or HCl to provide a pH = 7.2. For each virus, infective amnio-allantoic fluid was diluted 1:100 in the distilled water, filtered surface water, and intact surface water. Inoculated water samples were divided into 2 mL aliquots in 5 mL polystyrene round-bottom tubes and placed in incubators and maintained at a constant temperature of 17°C (n = 45 per each virus-water type combination). Virus-negative filtered and intact surface water controls were setup using the same methods as the experimental trials and run concurrently to ensure no environmentally deposited cytopathic agents including IA virus were present in the surface water samples (n = 45 per water type). All experiments and controls were run in triplicate.

For each water type, the TCID_50_/mL was determined at the time of inoculation (day 0) and 11–15 times post-inoculation with the sampling interval varying from 3–6 days depending on the water type. At each sampling, the tubes containing the virus inoculated water samples were removed from the incubator and vortexed to resuspend any particulate matter and thoroughly mix the suspension. Duplicate 0.5 mL aliquots were removed from each tube and diluted 1:1 by addition of 0.5 mL of 2X minimal essential medium (MEM). Ten-fold dilutions (10^-1^ to 10^-6^) were made in 1X MEM supplemented with antibiotics (10000 U/mL Penicillin G, 10 mg/mL Streptomycin, 25 μg/mL Amphotericin). Infectivity assays were performed on MDCK cells [8].

### Statistical analyses

Results from duplicate titrations were averaged and log_10_ transformed prior to analyses. Linear regressions were used to calculate the time required for a 90% reduction of infectivity in water (i.e. time required for a decrease of the viral titer by 1 log_10_ TCID_50_/mL [6,8]. Fligner–Killeen tests were used to check for homogeneity of variance [28]. An analysis of covariance (ANCOVA) was used to test the effects of time, virus strain and type of water on virus infectivity. All statistical analyses were carried out in R 2.12.1 (http://www.R-project.org).

## Competing interests

The authors declare that they have no competing interests.

## Authors’ contributions

SPK, CL and DES conceived the study. SPK and CL performed the experiments and analyzed the data. SPK, CL and DES wrote the paper. All authors read and approved the final manuscript.
